# Out-of-hospital cardiac arrests in Switzerland: Predictors for emergency department mortality in patients with ROSC or on-going CPR on admission to the emergency department

**DOI:** 10.1371/journal.pone.0188180

**Published:** 2017-11-16

**Authors:** Thomas C. Sauter, Nora Iten, Patrik R. Schwab, Wolf E. Hautz, Meret E. Ricklin, Aristomenis K. Exadaktylos

**Affiliations:** 1 Department of Emergency Medicine, Inselspital, University Hospital Bern, Bern, Switzerland; 2 Sanitaetspolizei Bern, Emergency Medical Service, Bern, Switzerland; Medizinische Hochschule Hannover, GERMANY

## Abstract

**Background:**

One of the leading causes of death is out-of-hospital cardiac arrest (OHCA) with an in-hospital mortality of about 70%. To identify predictors for the high mortality of OHCA patients and especially for women, that are considered at high risk for in-hospital mortality, we evaluated one specific setting of in-hospital treatment after OHCA: the emergency department (ED).

**Methods:**

Retrospective analysis of consecutive ED admissions with OHCA at the Inselspital Bern, Switzerland from 1^st^ June 2012 to 31^th^ Mai 2015. Demographic, preclinical and ED medical data were compared for patient groups with return of circulation (ROSC) and on-going resuscitation (CPR) on admission, as well as for subgroups with and without ED mortality. Predictors for ED mortality were investigated using univariate analysis with logistic regression.

**Results:**

In 354 patients (228 (64.4%) with ROSC; 126 (35.6%) with on-going CPR) we found an overall ED mortality of 28.5% (5.7% ROSC group; 69.8% on-going CPR group). Female gender (OR 7.053 (CI 95% 2.085; 24.853), p = 0.002) and greater age (OR 1.052 (95% CI 1.006–1.101), p = 0.029) were associated with ED mortality in the ROSC but not in the on-going CPR group. Ventricular fibrillation as initially monitored rhythm (OR 0.126 (95% CI 0.027–0.582), p = 0.008) and shorter CPR duration (OR 1.055 (95% CI 1.024;1.088), p = 0.001) were associated with ED survival in patients with ROSC but not in patients with on-going CPR on admission. In ROSC patients a higher lactate and lower pH were associated with mortality (pH: OR 0.009 (CI95% 0.000;0.420), p = 0.016; lactate: OR 1.183 (95% CI 1.037; 1.349), p = 0.013); similar in on-going CPR patients (pH 0.061 (95% CI 0.007, 0.558), p = 0.013, lactate: 1.146 (95% CI 1.041;1.261), p = 0.005).

**Conclusion:**

Patients with ROSC who died during ED care were predominantly women and older patients, as well as patients with non-shockable initial heart rhythm and long CPR durations. In patients with on-going CPR on admission, no clinical or demographic predictors for ED mortality were found. Higher lactate and lower pH were predictors in both OHCA groups.

## Introduction

One of the leading causes of death in Europe is out-of-hospital cardiac arrest (OHCA), with an overall survival rate of 2.6–9.9%[1, 2]. The EuReCa ONE study recently reported that 35.9% of patients arrived at the hospital after initiation of cardiopulmonary resuscitation (CPR) by the emergency medical service (EMS)—25.2% with return-of-circulation (ROSC) and 10.7% with on-going CPR)[3]. Large investigations in the US in 2006 and UK in 2007 reported in-hospital mortality of 67% and 71%[4, 5], respectively. Forty percent (40%) of patients with OHCA have an initial shockable rhythm (ventricular fibrillation/ventricular tachycardia), but only 22% of these patients achieve ROSC[6]. Resuscitation rules to predict OHCA outcome exist for EMS, but not for clinical settings such as the emergency department (ED) [7, 8]. It has been reported that witnessed arrest, bystander CPR, patients found in shockable rhythm and ROSC achieved in the field are predictors of survival on discharge from hospital[6].

Research on patient factors—such as gender—associated with in-hospital mortality is still on-going: It is known that women have a worse outcome after cardiac events in general[9]. Surprisingly, there is evidence that preclinical survival after OHCA until hospital admission is significantly better for women than men, which raises the question of why survival to discharge for women is worse[10]. The reason for the higher in hospital mortality for women is not completely understood and women are considered to be at high risk of in-hospital mortality[11].

Neumar et al. suggested that outcomes for pre-clinical, emergency department and discharge time points should be evaluated separately as protective and endangering factors may be different in the different phases, and interventions to improve the performance of the different care providers may need to account for these differences[12]. To better understand the significant in-hospital mortality of OHCA patients, and women in particular, we thus evaluated a specific setting of in-hospital treatment after OHCA: the emergency department.

We describe an ED population after OHCA in the German speaking part of Switzerland, Canton Berne and address the following research questions:

- What is the ED mortality for patients with ROSC and on-going CPR on ED admission?
- Are there differences between patient groups with or without ED mortality in patients with ROSC or with on-going CPR on ED arrival?
- Are there patient characteristics or clinical factors that are associated with ED mortality in patients with ROSC or on-going CPR on ED arrival?

## Methods

The present investigation included all admissions to the University Emergency Department (ED) Inselspital in Bern, Switzerland from 1st June 2012 to 31th Mai 2015. The ED has a catchment area of about 2 million people and treats about 45,000 patients per year[13]. Medical data from the electronic health record of the ED were screened for the keyword “resuscitation”, as well as common variations in spelling. Patients >18 years of age admitted after out-of-hospital cardiac arrest (OHCA) were included in further evaluations. We excluded traumatic cardiac arrest patients from our study due to the different characteristics of arrest. We collected demographic data (age, gender), medical data documented by the ambulance team (non-interventional time (downtime), first monitored rhythm, witnessed arrest, bystander CPR, medication given, number of shocks administered) and ED medical data (personal history, blood pressure and heart rate, oxygen saturation, GCS at admission, medication given, amount of shocks administered, ED mortality, blood results at admission, total duration of CPR and time to ROSC as well as extra corporal life support system (ECLS) at ED). In our hospital a standardized protocol for ECLS in patients with on-going CPR at ED arrival is implemented. Mandatory preconditions for initiation of ECLS defined at our institution are age <65 years and begin of ECLS <45 minutes after start of CPR. The final decision about ECLS is made by the treating ED team.

We used the updated Utstein style definitions for return of spontaneous circulation (ROSC), bystander CPR, witnessed arrest, first monitored rhythm and shockable rhythm[14].

Statistical analysis was performed with SPSS Statistics 21 (IBM Zurich, Switzerland). Descriptive statistics were used to describe the baseline characteristics of the patients. Patient demographic data, patient flow data, medical data (both preclinical and in the emergency room) were compared in patient groups with ROSC or with on-going CPR on admission, as well as subgroups with and without ED survival, using independent sample t tests or the Mann-Whitney U test depending on the normality of the data, or Pearson’s chi-square test or Fishers exact tests, as applicable. Statistical significance was defined as a p value <0.05. Association of different parameters with ED mortality in resuscitation patients were assessed in univariate and multivariate binary logistic regression. The present investigation is registered with the Ethics Committee of the Canton Bern, Switzerland (ReQ-2017-00128), that waived the need for full ethical review or patient consent.

## Results

**Fig 1:**
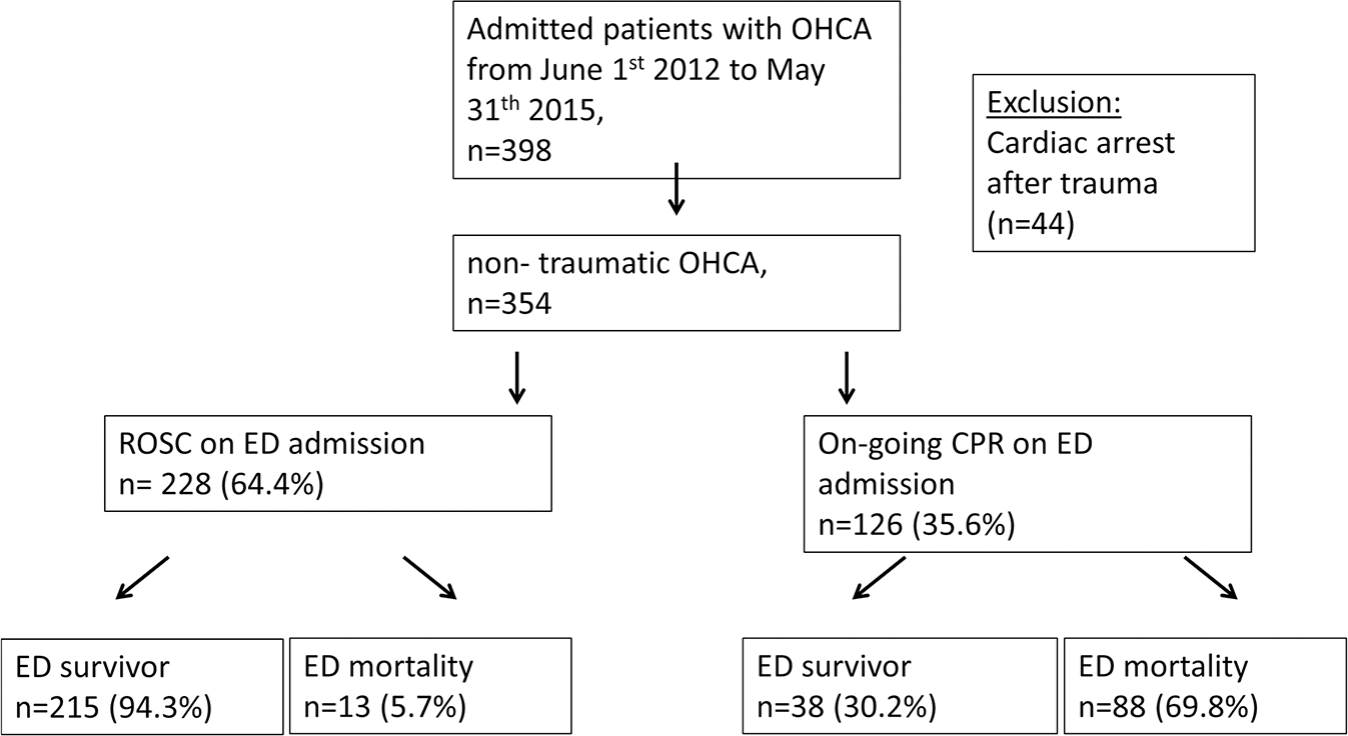
Flowchart. Patient flowchart.

Our database search over 3 years returned 398 patients with OHCA admitted to our hospital (Fig 1). After excluding 44 patients with traumatic cardiac arrest, we included 354 patients (228 patients (64.4%) with ROSC and 126 patients (35.6%) with on-going CPR on admission). We found an overall ED mortality of 28.5% (5.7% in the ROSC group and 69.8% in the on-going CPR group) in patients admitted after OHCA.

The baseline characteristics of OHCA patients are summarised in Table 1. The groups with ROSC or on-going CPR were comparable with respect to age and gender. The numbers of witnessed cardiac arrests and bystander CPR were equal in the two groups (number of patients with witnessed cardiac arrests: 152 (66.7%) ROSC patients vs. 84 (66.7%) on-going CPR patients, p = 0.678; number of patients with bystander CPR: 148 (41.8%) ROSC patients vs. 53 (42.1%) on-going CPR patients, respectively).

**Table 1:** Baseline characteristics of OHCA patients according to ROSC or ongoing CPR on admission.

Characteristics	OHCA (n = 354)	ROSC on admission (n = 228)	Admission with ongoing CPR (n = 126)	p
Age (years)	64.9±15.9	65.6±15.5	63.6±16.7	0.269
Gender (female)	92 (26.0%)	61 (26.6%)	31 (24.6%)	0.659
History of ischaemic heart disease	85% (24%)	55 (24.1%)	30 (23.8%)	0.563
*Preclinical characteristics*				
First monitored rhythm (**asystole, ventricular fibrillation**, PEA, unknown)	**75 (21.2%); 171 (48.3%);** 77 (21.8%); 31 (8.6%)	**41 (18.0%); 129 (56.6%);** 44 (19.3%; 14 (6.1%)	**34 (27.0%); 54 (42.9%);** 33(26.2%); 5 (4.0%)	0.047*; 0.013*; 0.132
Witnessed arrest	236 (66.7%)	152 (66.7%)	84 (66.7%)	0.678
**Non-interventional interval (downtime) (min)**	**8.26±7.9**	**7.5±7.6**	**9.6±8.2**	0.019*
Bystander CPR	148 (41.8%)	95 (41.7%)	53 (42.1%)	0.845
Time to ROSC (min)	**23.1 (±18.2)**	**21.0 (±15.3)**	**36.3 (±27.3)**	0.001*
CPR duration (min)	**34.8 (±29.6)**	**20.5 (±16.6)**	**60.1 (±30.8)**	<0.001*
**Adrenaline administered (number)**	**304 (85.9%)**	**182 (79.8%)**	**122 (96.8%)**	<0.001*
**Adrenaline dosage (mg)**	**3.83±3.9**	**2.75±3.2**	**5.64±4.3**	<0.001*
Cordarone (number)	121 (34.2%)	79 (34.6%)	42 (33.3%)	0.803
Number of shocks	2.08±2.8	1.87±2.5	2.46±3.5	0.649

n = 354, Mean ± standard deviation or percentage (%), independent samples t test, Mann-Whitney U, Pearson’s Chi-Square or Fisher's exact test as applicable, p<0.05*

ROSC = Return of spontaneous circulation; CPR = cardiopulmonary resuscitation; PEA = pulseless electrical activity, OHCA = out of hospital cardiac arrest; ED = emergency department.

The non-interventional time was significantly lower in the ROSC group than in the on-going CPR group (min: 7.5±7.6 vs. 9.6±8.2, p = 0.019). The time to ROSC as well as the overall CPR duration were significantly longer in the on-going CPR group in comparison with the ROSC group (Time to ROSC: 21.0±15.3 vs. 36.3±27.3 min, p = 0.001, CPR duration 20.5±16.6 vs. 60.1±30.8 min, p<0.001, respectively).

Asystole was documented in more patients with on-going CPR than in patients with ROSC on admission (number of patients with asystole: 41 (18.0%) ROSC group vs. 34 (27.0%) on-going CPR group, p = 0.047). In contrast, ventricular fibrillation was found in fewer patients with on-going CPR than in patients with ROSC on admission (129 (56.6%) ROSC group vs. 54 (42.9%) on-going CPR group, p = 0.013, respectively).

### Comparison of OHCA patients with ROSC on arrival at the ED with respect to ED mortality

Patients with ED mortality were significantly older than patients who survived the ED (years: 75.1±13.1 vs. 65.0±15.5, p = 0.022). Significantly more female patients with ROSC died in the ED than men (female: 52 (24.2%) vs. 9 (29.2%), p = 0.001). Significantly fewer preclinical shocks were administered to patients with ED mortality (number of shocks: 0.4±0.7 vs. 1.94±2.4, p = 0.011). The only significant difference in the initially monitored heart rhythm was the significantly lower incidence of ventricular fibrillation in patients with ED mortality (ventricular fibrillation: 2 (16.7%) vs. 127 (59.1%), p = 0.002).

Time to ROSC was not significantly different in both groups (20.4±14.7 vs. 31.1±22.7 min, p = 0.139). The CPR duration was found to be significantly longer in the mortality group compared to patients with ED survival (41.8±18.9 vs. 19.3±15.6 min, p = 0.002).

Comparing blood results at admission, potassium and lactate were significantly higher (potassium: 5.6±2.2 vs. 4.2±0.8 mmol/l, p = 0.034, lactate 11.2±5.1 vs. 6.1±4.4 mmol/l, p = 0.013), the pH significantly lower in the non-survival group compared to the survival group (7.019±0.136 vs. 7.200±0.178, p = 0,010; see Table 2). For a summary of all compared parameters see Table 2.

**Table 2:** Comparison of OHCA patients with ROSC on arrival at ED regarding ED mortality.

Parameter	ED survival (n = 215)	ED non survival (n = 13)	p
**Age (years)**	**65.0±15.5**	**75.1±13.1**	0.022*
**Gender (female)**	**52 (24.2%)**	**9 (69.2%)**	0.001*
*Preclinical data*			
Non-interventional interval (downtime) (min)	7.2±7.1	13.5±14.9	0.285
First monitored rhythm (asystole, **ventricular fibrillation,** PEA, unknown)	36(16.7%); **127 (59.1%);** 39 (18.1%); 13 (6.0%)	5 (38.5**%); 2 (16.7%),** 5 (38.5%); 1 (7.7%)	0.062; **0.002;** 0.138
Witnessed arrest	146 (67.9%)	6 (46.2%)	0.176
Bystander CPR	91 (42.3%)	4 (30.8%)	1.000
**Preclinical number of shocks**	**1.94±2.4**	**0.4±0.7**	0.011*
Time to ROSC (min)	20.4±14.7	31.1±22.7	0.139
**CPR duration (min)**	**19.3±15.6**	**41.8±18.9**	0.002*
*Medication*:			
Preclinical adrenaline given	169 (78.6%)	13 (100%)	0.076
**Preclinical adrenaline (mg)**	**2.6±3.1**	**5.4±3.5**	0.016*
Preclinical amiodarone given	77 (35.8%)	2 (15.4%)	0.228
Preclinical atropine given	49 (22.8%)	2 (15.4%)	0.738
*Emergency department data*			
Clinical adrenaline administered (number)	49 (22.8%)	6 (46.2%)	0.088
Clinical number of shocks	0.06±0.29	0±0	.454
Diagnosis of ST-elevation myocardial infarction	58 (27.8%)	2 (15.4%)	1.000
**Coronary angiography performed**	**158 (73.5%)**	**0 (0%)**	<0.001*
Diagnosis of pulmonary embolism	7 (3.3%)	1 (7.7%)	0.380
History of CHD	50 (23.3%)	5 (38.5%)	0.347
GCS	4.4±3.6	3.2±0.83	0.356
**Systolic blood pressure (mmHg)**	**114.7±43.7**	**50.6±46.5**	<0.001*
**Diastolic blood pressure (mmHg)**	**65.63±28.5**	**35.3±32.4**	<0.001*
**Heart rate (bpm)**	**89.8±28.3**	**41.1±51.7**	0.031*
Oxygen saturation (%)	94.9±11.0	92.0±2.7	0.649
*Blood results at admission*			
**Potassium (mmol/l)**	**4.2±0.8**	**5.6±2.2**	0.034*
**pH**	**7.200±0.178**	**7.019±0.136**	0.010*
**Lactate (mmol/l)**	**6.1±4.4**	**11.2±5.1**	0.013*
Creatinine (μmol/l)	121.4±55.8	129.7±30.6	0.720
Haemoglobin (g/l)	132.6±22.9	122.3±26.3	0.254

Mean ± standard deviation or absolute numbers (%), independent samples t test or Mann-Whitney U, Pearson’s chi-Square test or Fishers exact test as applicable, p<0.05*.

CPR: cardiopulmonary resuscitation, ED: emergency department, ROSC: return of spontaneous circulation, ECLS: extra corporal life support.

### Comparison of OHCA patients with on-going CPR on arrival at ED with respect to ED mortality

For patients with on-going CPR on arrival at the ED, survival were not associated with age or gender (age in years: 60.3±16.4 vs. 65.0±16.7; p = 0.151; male gender: 29 (76.3%) vs. 66 (75.0%); p = 0.875). Moreover, there was no difference in the initially monitored heart rhythm. Neither time to ROSC nor overall CPR duration were significantly different in the compared patient groups (Time to ROSC: 36.8±24.4 vs. 34.8±36.6 min, p = 0.888; CPR duration: 53.1±30.2 vs. 62.9±30.8 min, p = 0.111, respectively).

Regarding blood results, the pH was found to be significantly lower (7.057±0.213 vs. 6.911±0.245, p = 0.009), lactate significantly higher (9.9±5.2 vs. 14.2±6.0 mmol/l, p = 0.003) at admission in the mortality group compared to patients with ED survival. For a summary of all compared parameters see Table 3.

**Table 3:** Comparison of OHCA patients with ongoing CPR on arrival at ED regarding ED mortality.

Parameter	Survival (n = 38)	Non survival (n = 88)	p
Age (years)	60.3±16.4	65.0±16.7	0.151
Gender (female)	9 (23.7%)	22 (25.0%)	0.875
*Preclinical data*			
Non-interventional interval (downtime) (min)	11.1±9.3	9.2±7.8	0.294
First monitored rhythm (asystole, ventricular fibrillation, PEA, unknown)	11 (28.9%); 17 (44.7%); 10 (26.3%); 0 (0%)	23 (26.1%); 37 (42.0%); 23 (26.1%); 5 (5.7%)	0.744; 0.779; 0.983
Witnessed arrest	24 (63.2%)	60 (68.2%)	0.357
Bystander CPR	14 (36.8%)	39 (44.3%)	0.270
Preclinical number of shocks	2.8±3.7	2.3±3.4	0.444
Time to ROSC (min)	36.8±24.4	34.8±36.6	0.888
CPR duration (min)	53.1±30.2	62.9±30.8	0.111
*Medication*:			
Preclinical adrenaline given	37 (97.4%)	85 (96.6%)	1.000
Preclinical adrenaline (mg)	5.1±3.1	5.9±4.7	0.431
Preclinical amiodarone given	14 (36.8%)	28 (31.8%)	0.583
Preclinical atropine given (number)	5 (13.2%)	12 (13.6%)	0.942
*Emergency department data*			
Clinical adrenaline (number)	37 (97.4%)	88 (100%)	0.302
Clinical number of shocks	0.7±1.7	0.3±1.0	0.458
Diagnosis of ST-elevation myocardial infarction	7 (18.4%)	3 (3.4%)	0.725
Diagnosis of pulmonary embolism	2 (5.3%)	8 (9.1%)	0.371
History of CHD	7 (18.4%)	23 (26.1%)	0.260
ECLS	3 (7.9%)	2 (2.3%)	0.161
*Blood results at admission*			
Potassium (mmol/l)	4.3±1.0	4.9±1.5	0.083
**pH**	**7.057±0.213**	**6.911±0.245**	0.009*
**Lactate (mmol/l)**	**9.9±5.2**	**14.2±6.0**	0.003*
Creatinine (μmol/l)	149.7±122.8	141.5±54.5	0.741
Haemoglobin (g/l)	135.0±21.5	132.3±25.8	0.638

Mean ± standard deviation or absolute numbers (%), independent samples t test or Mann-Whitney U, Pearson’s chi-Square test or Fishers exact test as applicable, p<0.05*.

CPR: cardiopulmonary resuscitation, ED: emergency department, ROSC: return of spontaneous circulation, ECLS: extra corporal life support.

### Association of parameters with ED mortality in OHCA patients in univariate analysis

The associations of different parameters with ED mortality were investigated by univariate analysis for patients with ROSC (Table 4) and patients with on-going CPR at arrival (Table 5).

**Table 4:** Univariate analysis of factors associated with ED mortality in OHCA patients with ROSC on arrival at ED.

Parameter	p	Odds ratio (confidence interval)
Age	0.026*	1.052 (1.006;1.101)
Female gender	0.002*	7.053 (2.085;24.853)
Ventricular fibrillation	0.008*	0.126 (0.027;0.582)
Number of preclinical shocks	0.049*	0.421 (0.178–0.997)
Amount of preclinical adrenaline	0.027*	1.193 (1.020–1.395)
CPR duration	0.001*	1.055 (1.024;1.088)
Systolic blood pressure	<0.001*	0.966 (0.951;0.982)
Diastolic blood pressure	<0.001*	0.947 (0.922; 0.974)
Heart rate	<0.001*	0.964 (0.944; 0.984)
Potassium	0.003*	2.444 (1.352;4.417)
pH	0.016*	0.009 (0.000;0.420)
Lactate	0.013*	1.183 (1.037;1.349)

Binary logistic regression, p<0.05*, 95% Confidence interval.

**Table 5:** Univariate analysis of factors associated with ED mortality in OHCA patients with ongoing CPR on arrival at ED.

Parameter	p	Odds ratio (confidence interval)
pH	0.013*	0.061 (0.007;0.558)
Lactate	0.005*	1.146 (1.041;1.261)

Binary logistic regression, p<0.05*, 95% Confidence interval.

Female gender was strongly associated with ED mortality in patients with ROSC on admission (OR 7.053 (95% CI 2.085; 24.853), p = 0.002). Moreover, higher age was associated with ED mortality (OR 1.052 (95% CI 1.006–1.101), p = 0.029). A greater number of preclinical shocks (OR 0.421 (95% CI 0.178–0.997)) and a higher amount of preclinical adrenaline given were found to be associated with ED mortality in patients with ROSC on arrival (OR 1.193 (95% CI 1.020–1.395); all p<0.05). Ventricular fibrillation as initially monitored rhythm (OR 0.126 (95% CI 0.027–0.582)) was associated with ED survival in patients with ROSC on admission (p = 0.008), but not in patients with on-going CPR on admission. Higher potassium (OR 2.444 (95% CI 1.352;4.417), p = 0.003) and higher lactate (OR 1.183 (95% CI 1.037;1.349), p = 0.013) as well as lower pH (OR 0.009 (95% CI 0.000;0.420), p = 0.016) were associated with mortality in the ROSC group; lower pH (OR 0.013 (95% CI 0.061;0.558), p = 0.013) and higher lactate (OR 1.146 (95% CI 1.041;1.261), p = 0.005) in the on-going CPR group. Multivariate testing of parameters identified as predictors in univariate analysis was not possible because a large number of patients did have missing values in the predictors identified in univariate modelling.

## Discussion

Our study found an ED mortality of 28.5% after OHCA (5.7% for patients admitted with ROSC and 69.8% for patients with on-going CPR on admission). More women and elderly patients subsequently died in the patient group with ROSC but not in the patient group with on-going CPR on ED admission. Female gender was found to be strongly associated with ED mortality in patients with ROSC on ED admission after OHCA. Furthermore, our study suggests that ventricular fibrillation as initially monitored rhythm is associated with ED survival in patients with ROSC on ED arrival but not in patients with on-going CPR on admission.

Other studies have shown that the in-hospital mortality of patients admitted after OHCA is about 2/3. We now show that much of this mortality occurs in the ED[4, 5]. For ED clinicians it is important to keep in mind that even the patient group that arrives at the ED with ROSC after successful pre-clinical resuscitation has an ED mortality of 5.7%. The high mortality (nearly 70%) in patients admitted to our ED department with on-going CPR may partially be explained by local EMS conditions: In our catchment area, not all EMS services are legally allowed to terminate CPR in the preclinical setting without consultation of a physician but have to transport the patient to hospital care. As result of this, patients may be admitted to hospital that would not have been transported in other settings. Another factor contributing to the high mortality in this group is the longer documented downtime, that is known to be associated with a poor outcome[6, 15]. It is interesting, that in the ROSC at admission group CPR duration was nearly double length in patients with ED mortality compared to patients with ED survival. It may be an important point for any ED physician to notice that patients admitted with ROSC with a long duration of CPR are at higher risk of death in contrast to patients with long and on-going CPR. Although there is a standardized protocol for ECLS implemented at our ED, the number of patients eligible for ECLS was found to be very low, maybe due to the strict inclusion criteria as mentioned above.

The on-going discussion about female gender as risk factor for mortality is also an issue in our specific ROSC population. Even with the conservative procedure of using the lower limits of the 95% confidence interval of the odds ratio, twice the number of female patients that in our ROSC population die than men. However, the significantly greater mortality of female ROSC patients could not be demonstrated in patients with on-going CPR on admission. It has been speculated that the higher in-hospital mortality in women might result from women being less likely to fulfil Utstein event characteristics associated with survival. Age modified the association of gender and mortality, with increased mortality in postmenopausal women[16]. Therefore, there may be a protective effect of hormones in premenopausal women. Hasan et al. found a greater total in-hospital mortality for women and speculated about possible reasons, e.g. hormonal factors, reaction of the vegetative nerve system to cardiac events or differences between men and women in the aetiology of cardiac arrest. Our data do not permit any conclusion about the factors influencing the higher female mortality in our specific ED ROSC population. Ventricular fibrillation was more frequent in our ROSC group with ED survival, which is consistent with the idea that survival in this group is related to cardiac factors and that the aetiology of cardiac arrest may differ between men and women. It is well documented that patients with a shockable rhythm have increased overall survival[6]. This is consistent with our finding that a prehospital shockable initial heart rhythm was associated with ED survival in patients with ROSC on admission, but this was not the case for our patient group of patients with on-going CPR on admission.

Patients with ROSC on ED admission who did not survive during the ED treatment were found to be significantly older than patients with ED survival. This association of increased age with mortality could not be demonstrated in the patient group with on-going CPR on admission. Although it may be suspected that there are greater inhibitions to terminate CPR in younger patients, the patients group with on-going CPR were not significantly younger than the ROSC group. Our study suggests that emergency physicians responsible for the treatment of resuscitated patients and who present with ROSC should bear in mind that enhanced caution is necessary for the treatment of older women with non-shockable initial heart rhythm. In contrast to this, neither the initial heart rhythm, nor gender nor age were found to be predictive for ED outcome in patients brought to the ED with on-going CPR on admission.

The elevated lactate and lower pH, found in both groups to be predictive of mortality, are surrogate parameters for metabolic acidosis most likely caused by prolonged impaired perfusion. As there is a lack of known clinical predictors or patient predictors for ED outcome, blood tests may help to guide the ED clinician.

### Limitations

As our study is a retrospective evaluation involving pre-existing medical records, no guarantee can be given that all eligible patients were correctly reported. Careful checks of the results found in the medical database were performed to reduce biases. Furthermore, our study is a single centre study and therefore further research about the transferability to other populations is desirable. Given the high socio-economic impact of OHCA, structured prospective evaluation of all OHCA patients to improve quality of data is necessary—not only in the preclinical setting but also in the ED. A structured largescale registry would give the opportunity to confirm the identified predictors and enable multivariate testing. Unfortunately, there is no registry of OHCA patients in the German speaking part of Switzerland. In the European Registry EuReCa, only Canton Ticino, the Italian speaking part of Switzerland, is represented (only about 4% of the population of Switzerland)[2].

## Conclusion

Our study gives an insight into the predictors of ED mortality of an OHCA arrest population in the German part of Switzerland. As the 5.7% of patients with ROSC who died during ED care were predominantly women, older patients, patients with long CPR durations and patients with non-shockable initial heart rhythm, special attention should be paid to these patient groups. In patients with on-going CPR on admission, no clinical or patient predictors were found for the demonstrated 69.8% ED mortality. In both patient groups blood results may help the clinician as pH as well as lactate were predictors in both compared patient groups.
